# Digestion kinetics of carbohydrate fractions of citrus by-products

**Published:** 2015-03-15

**Authors:** Saman Lashkari, Akbar Taghizadeh

**Affiliations:** 1 *Department of Animal Science, Faculty of Agriculture, University of Kurdistan, Sanandaj, Kurdistan, Iran; *; 2 *Department of Animal Science, Faculty of Agriculture, University of Tabriz, Tabriz, Iran.*

**Keywords:** Carbohydrate fractions, Citrus by-products, Digestion kinetics, Gas production

## Abstract

The present experiment was carried out to determine the digestion kinetics of carbohydrate fractions of citrus by-products. Grapefruit pulp (GP), lemon pulp (LE), lime pulp (LI) and orange pulp (OP) were the test feed. Digestion kinetic of whole citrus by-products and neutral detergent fiber (NDF) fraction and acid detergent fiber (ADF) fractions of citrus by-products were measured using the *in vitro* gas production technique. Fermentation kinetics of the neutral detergent soluble carbohydrates (NDSC) fraction and hemicelluloses were calculated using a curve subtraction. The fermentation rate of whole was the highest for the LE (*p* < 0.05). For all citrus by-products lag time was longer for hemicellulose than other carbohydrate fractions. There was no significant difference among potential gas production (*A*) volumes of whole test feeds (*p *< 0.16). Dry matter (DM) digestibility contents of LE and LI were the highest (*p* < 0.02). The NDF digestibility was the highest (*p* < 0.05) in LI and GP, while the lowest (*p* < 0.03) values of ADF digestibility were observed in LI and LE. According to the results of the present study, carbohydrate fractions of citrus by-products have high potential for degradability. It could also be concluded that carbohydrate fractions of citrus by-products have remarkable difference in digestion kinetics and digestive behavior.

## Introduction

Citrus pulp is a by-product feed obtained during the industrial orange juicing and processing of other citrus fruits.^1^ Citrus by-products have high potential rumen degradability and high apparent total tract digestibility. Citrus pulp can be fed fresh, ensiled or dried. It has been successfully fed to dairy cattle and fattening lamb.^2^^,^^3^

*In vitro* digestion rates from gas curves can be used in nutritional models. The beef NRC and cornel net carbohydrate and protein system include a nutrition model that requires digestion rates of fiber and soluble fiber fractions.^4^^,^^5^ The digestion of neutral detergent fiber (NDF) and acid detergent fiber (ADF) fractions can be measured using *in vitro* methods because these feed fractions can be chemically isolated.^6^ There was little information about digestion kinetics of neutral detergent soluble carbohydrates (NDSC) and most of data for NDSC obtained from *in vitro* disappearance of insoluble cell wall content. Schofield and Pell used an approach to study the digestion kinetics of NDSC by subtraction method.^7^ Amount of gas production of NDSC is obtained by the difference in volumes of gas produced between the whole feed and it’s respective NDF fraction.^8^ However, gas production of hemicelluloses is calculated by the difference in volumes of gas produced between the NDF and ADF fraction.^8^ This curve subtraction approach, allows to evaluate digestion behavior of fractions that may be difficult to extract completely or without degradation. It also can omit the associative effects of other feed fractions on the fermentation kinetics of the carbohydrate fraction.^6^

 The purpose of this study was to determine digestibility and fermentation characteristics of whole, NDF and ADF fractions of four citrus by-products using *in vitro* gas production. Fermentation characteristics of hemicellulose and NDSC fermentation include fermentation rates, lag time and gas production volumes were calculated using curve subtraction.

## Materials and Methods

**Sample collection. **This research project was conducted in Agriculture Research Station of University of Tabriz, Iran. Fresh orange pulp (OP), lime pulp (LI), lemon pulp (LE) and grapefruit pulp (GP) were collected without any processing after mechanical extraction. Six different batches per type of pulp were sampled, then all samples were thoroughly mixed, and a composite sample was taken for chemical analysis, carbohydrate fraction extraction and *in vitro* gas production. Citrus by-products were obtained from a main factory in Tabriz, Iran. 

**Chemical composition. **Feedstuffs dry matter (DM, Method ID 934.01), ash (Method ID 942.05), ether extract (EE, Method ID 920.30), and crude protein (CP, Method ID 984.13) were determined by procedures of AOAC.^9^ Neutral detergent fiber (NDF) and acid detergent fiber (ADF) were determined using an ANKOM^200/220^ Fiber Analyzer (Ankom Technology, Macedon, USA) According to the manufacturer’s instructions without sodium sulphite.^10^ Pectin content of citrus by-products was determined according to the method described by Kratchanova *et al*.^11^

**Preparation of isolated NDF and ADF fractions. **The NDF was isolated according to the method recommended by Schofield and Pell.^7^ Briefly, the NDF fraction was extracted by autoclaving several bottles containing 2.40 g of citrus by-products and solution containing 100 mL of neutral detergent and 0.40 mL of termamyl at 105 ˚C for 1 hr. Then the pooled extracted fiber was rinsed several times with hot distilled water using a 37 mm mesh nylon screen as a filter and then rinsed the fiber with 100 mL each of ethanol and acetone. The fiber was subjected to vacuum until it was almost dry and was then incubated overnight at 39 ˚C with 100 mL of 1 M ammonium sulfate. The filtration and wash repeated, and then the fiber dried in an oven at 50 ˚C for 48 hr. The ADF was isolated according to procedure of Darcy and Belyea.^12^ Briefly, the ADF fraction was extracted with acid detergent slution.^8^ The residual was boiled in neutral detergent solution for 1 hr for removal of residual quaternary ammonium ions of acid detergent solution. Finally, ADF was boiled in distilled water for 1 hr and filtered to remove traces of residual neutral detergent solution.

***In vitro*** **gas production. **Four 3-year-old ruminally fistulated Iranian *Ghizel* male sheep (average body weight 35.00 ± 3.00 kg) were used in this study. The overall health of the sheep was monitored prior to and throughout the study. The animals were housed in a ventilated barn in the individual metabolic cages and were acclimatized to the experimental conditions for 10 days. All experimental procedures were approved by the Advisory Committee of University of Tabriz Research Council. 

Individual samples of each citrus by-products (in six replicates) of the whole, NDF and ADF were incubated in 50 mL serum bottles according to the procedure of Fedorak and Hurdy in three separate runs.^13^ Ruminal fluid was obtained approximately 2 hr after the morning feeding from four rumen cannulated sheep consuming 400 g per day alfalfa hay, 200 g barley grain per day, and 200 g per day soybean meal. The diet was offered twice a day at 08:30 and 15:30 hr in equal amounts after collecting the refusals. All animals were given free access to mineral salt lick and water throughout the experiment. Equal volumes of ruminal fluid (about 350 mL per sheep) from each sheep collected and combined. Rumen fluid was pumped with a manually operated vacuum pump and transferred into pre-warmed thermos flask, filtered through four layers of cheesecloth and flushed with CO_2_ and was transported to the laboratory in sealed thermos. The resulting ruminal fluid was purged with deoxygenated CO_2 _before using it as the inoculum. All samples were then ground to pass through a 1-mm screen in a Wiley mill (Model 4, Arthur H. Thomas Co., Philadelphia, USA) before incubation. Approximately, 300 mg of dried by-products sample were weighed and placed into serum bottles. Twenty mL buffered rumen fluid with McDougall’s buffer was pipetted into each 50 mL serum bottle.^14^ The gas production was recorded after 2, 4, 6, 8, 12, 16, 24, 36, 48, 72 and 96 hr of incubation. Total gas values were corrected for the blank incubation, and reported gas values are expressed in mL per 100 mg of DM. The gas production profiles in triplicate were fitted to the following equation using a non-linear procedure^15^:

*y = A (1−e* ^−c(t−L)^ *)*

where, *Y* is the volume of gas production (mL per 100 mg DM) at time t, *A* is gas production from soluble and insoluble fraction, *c* is rate of gas production, *L* the lag time (hr) and *t* is the incubation time (hr). 

In order to determine digestibility, whole, NDF and ADF fractions were incubated in 50 mL serum bottles in three separate runs.^10^ Vials were removed after 48 hr of incubation and their contents were filtered through monofilament polyester cloth with pore size of 53 μm. The residues were washed three times by resuspension in phosphate buffer (pH = 7.4) followed by centrifugation (2500 rpm, 10 min, 4 ˚C). The residues were dried at 60 ˚C for 48 hr and weight was recorded. 

**Subtraction curve. **Estimation of the digestion rates for the NDSC and hemicellulose fraction was conducted according to the method described by Doane *et al*.^16^ Estimation of the digestion rates for the NDSC and hemicellulose fraction by curve subtraction requires that gas volumes produced by the separate preparations (NDF and ADF) be adjusted to a common basis in proportion to the content of each fraction within the whole tested feed.^13^ This adjustment was done by using 100 mg whole test feed DM as the basis. 

**Statistical analysis. **Data on *in vitro* ruminal fermentation parameters and digestibility of whole and carbohydrate fractions of each of three runs within sample were averaged. Mean values of each individual sample within citrus pulp species were the experimental unit.^17^^,^^18^

Experimental data were analyzed according to completely randomized design using GLM procedure of SAS (Version 6; SAS Institute, Carry, USA). Data of each of the three runs within the same pulp sample were averaged prior to statistical analysis. Data were analyzed using the following model:

*y*_ij_ *= μ + T+ e*_ij_

where, *y*_ij_ is dependent variable representing the response for i treatment; *μ* is mean; *T* is treatment and *e*_ij_ is residual. The means were compared using Duncan mean comparison test.

## Results

**Chemical composition. **Chemical composition of the citrus by-products was shown in Table 1. There was no significant difference among carbohydrate (CHO) content of citrus by- products (*p *< 0.06). Pectin content of GP and LE were the highest and the lowest, respectively (*p* < 0.001). Crude protein content was similar among types of citrus by-products (*p *< 0.09). The amount of ash was the highest for LI (*p *< 0.001). The amount of NDF was the lowest for OP (*p *< 0.001). 

**Specific rates of digestion and lag times. **The lag time of whole LE and GP were the highest (*p *< 0.004, Table 2). For NDSC fraction, the highest lag time was observed for LE (*p *< 0.004). For NDF fraction, the highest lag time was observed for GP (*p *< 0.003). For ADF fraction, lag time was the highest and the lowest for GP and OP, respectively (*p *< 0.001). The lowest lag time was observed for OP hemicelluloses (*p *< 0.002). Specific rate of whole (c) was the highest (*p *< 0.003) in LP .

For GP, LI and OP, specific rate of NDSC were higher than whole sample (Table 3, *p *< 0.001), however, specific rate of NDSC and whole sample were similar in LE. For GP, specific rate of hemicelluloses were lower than the other carbohydrate fractions. For LI, specific rate of ADF was lower than the other fractions. The ranking of total gas volume of all citrus by-products in ascending order were whole sample, NDSC, NDF, ADF and hemicelluloses. For GP, LI and OP, lag time of ADF were lower than the other carbohydrate fractions (*p *< 0.001). 

**Table 1:** Chemical composition of the citrus by-products (g per kg DM).

**Citrus by-products**	**DM **	**CHO** 1	**Pectin**	**CP**	**Ash**	**EE**	**NDF**	**ADF**	**H** **C** 2
**Grapefruit pulp**	909.14^a^	826.13	384.90^a^	91.40	58.69^b^	23.77^c^	166.58^b^	130.88^b^	35.69
**Lemon pulp**	871.01^c^	815.09	276.80^c^	95.40	54.58^b^	34.46^a^	166.85^b^	151.30^a^	15.55
**Lime pulp**	905.15^a^	809.72	312.76^b^	81.60	81.20^a^	27.47^b^	174.92^a^	145.32^a^	29.59
**Orange pulp**	893.30^b^	825.38	310.53^b^	85.03	55.11^b^	34.46^a^	147.43^c^	119.50^c^	27.93
**SEM**	4.589	2.719	12.145	2.15	3.36	1.44	3.10	4.02	2.85
***p*** **-value**	0.001	0.06	0.001	0.09	0.001	0.001	0.001	0.008	0.052

1 Carbohydrate calculated as 1000 – (Crude protein + Ash + Ether extract).

2 Hemicellulose calculated as NDF – ADF.

abc Different superscripts indicate significant differences within each column (*p* < 0.05).

**Table 2:** Total gas production volume, specific rates of digestion, lag phase and digestibility of the whole, neutral detergent soluble carbohydrate, neutral detergent fiber, acid detergent fiber, hemicellulose in citrus by-products

	**Total gas production volume ** **(mL per 100 mg DM)**	**Specific rates of digestion ** **(per hr)**	**Lag phase ** **(hr)**	**Digestibility** **(g per 100 g DM)**
**Whole (based on DM)**				
**Grapefruit pulp**	44.39	0.068^b^	1.03^ab^	86.60^ab^
**Lemon pulp**	43.60	0.065^b^	1.06^a^	89.50^a^
**Lime pulp**	44.04	0.095^a^	0.43^c^	89.70^a^
**Orange pulp**	40.96	0.053^b^	0.70^bc^	84.10^b^
**SEM**	0.60	0.005	0.086	0.856
***p*** **-value**	0.160	0.003	0.004	0.020
**Neutral detergent soluble carbohydrate** 1				
**Grapefruit pulp**	39.63	0.077^bc^	1.13^ab^	-
**Lemon pulp**	37.35	0.081^b^	1.33^a^	-
**Lime pulp**	40.21	0.108^a^	0.56^c^	-
**Orange pulp**	35.76	0.062^c^	0.93^b^	-
**SEM**	0.77	0.554	0.09	-
***p*** **-value**	0.131	0.001	0.004	
**Neutral detergent fiber**				
**Grapefruit pulp**	5.36^c^	0.026^a^	1.93^a^	84.60^ab^
**Lemon pulp**	14.26^a^	0.007^d^	0.40^c^	83.10^b^
**Lime pulp**	5.38^c^	0.018^b^	0.53^bc^	86.70^a^
**Orange pulp**	8.12^b^	0.011^c^	0.96^b^	82.60^b^
**SEM**	1.09	0.002	0.19	0.58
***p*** ** value**	0.001	0.001	0.003	0.022
**Acid detergent fiber**				
**Grapefruit pulp**	1.92^c^	0.046^a^	0.72^a^	72.10^a^
**Lemon pulp**	8.67^a^	0.007^c^	0.31^b^	68.10^b^
**Lime pulp**	4.16^b^	0.013^b^	0.30^b^	69.10^ab^
**Orange pulp**	8.01^a^	0.005^c^	0.21^c^	72.30^a^
**SEM**	0.87	0.005	0.05	0.69
***p*** **-value**	0.001	0.001	0.001	0.034
**Hemicellulose** 2				
**Grapefruit pulp**	3.73^b^	0.018^b^	3.63^a^	-
**Lemon pulp**	5.69^a^	0.007^c^	3.33^a^	-
**Lime pulp**	1.65^d^	0.028^a^	3.43^a^	-
**Orange pulp**	2.59^c^	0.020^b^	2.60^b^	-
**SEM**	0.45	0.002	0.12	-
***p*** **-value**	0.001	0.001	0.002	-

1 determined by subtraction method (whole minus neutral detergent fiber).

2 determined by subtraction method (Neutral detergent fiber minus acid detergent fiber.

abcd Different superscripts indicate significant differences within each column (*p* < 0.05).

**Fig. 1:**
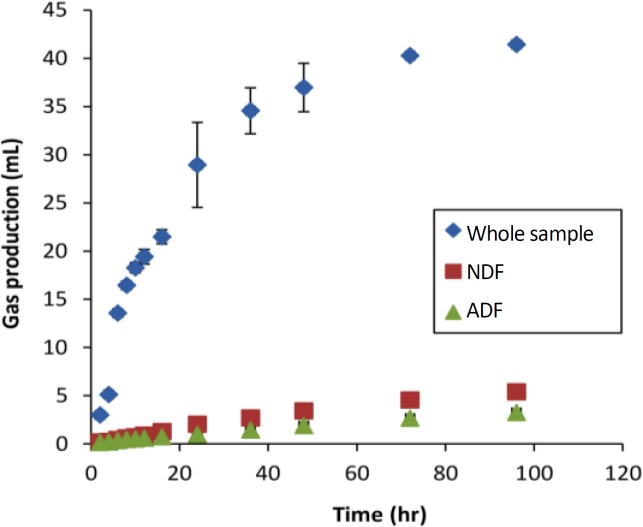
Gas production curves from the *in vitro* fermentation of orange pulp. Gas curves corrected to 100 mg DM and for the proportion of extracted residue in the whole sample. Error bars represent the standard errors.

**Fig. 2:**
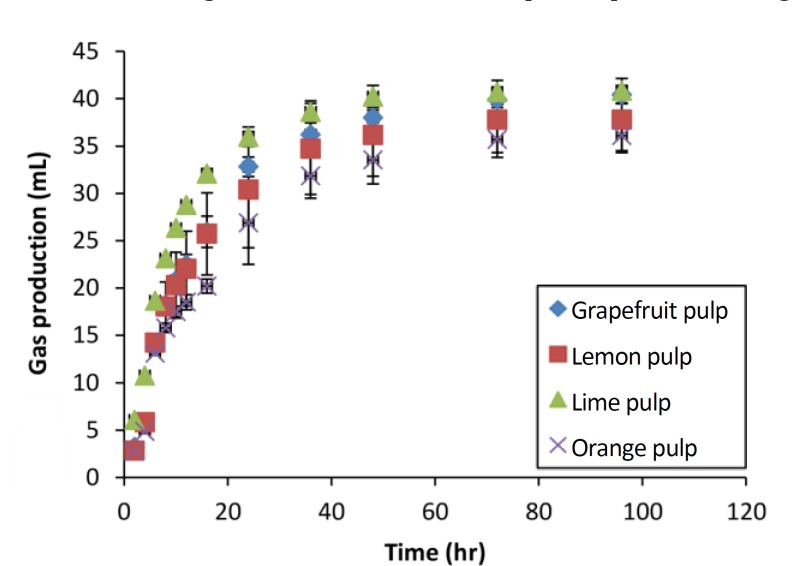
Subtracted gas production curves of neutral detergent-soluble from the *in vitro* fermentation of citrus by-products. Curves were calculated by subtracting the whole gas production from that of the natural detergent fiber at each observation time and are the average of all fermentations. Error bars represent the standard errors.

**Fig. 3:**
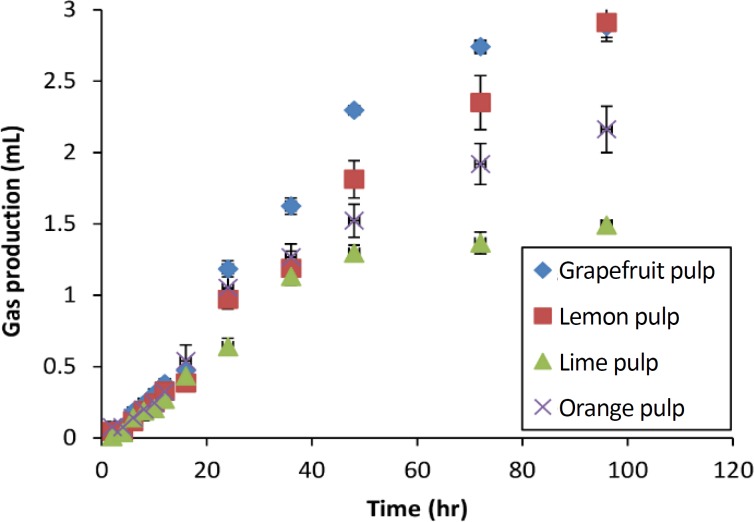
Subtracted gas production curves of hemicellulose from the *in vitro* fermentation of citrus by-products. Curves were calculated by subtracting the natural detergent fiber gas production from that of the acid detergent fiber at each observation time and are the average of all fermentations. Error bars represent the standard errors.

The gas curves of whole, NDF and ADF for the OP normalized to amount of the fraction studied contained in 100 mg DM, are shown in Figure 1. The NDSC and hemicelluloses gas production curves obtained by subtraction for citrus by-products are represented in Figures 2 and 3, respectively.

**Gas production and digestion data. **Potential gas production (A, mL per 100 m DM) and *in vitro* digestibility of the whole by-products, NDSC, NDF ADF and hemi-celluloses fractions of citrus by-products are shown in Table 2. There was no significant difference for potential gas production volume (A) among whole citrus by-products. Dry matter digestibility was the highest (*p *< 0.02) in whole LP and LE. Neutral detergent fiber digestibility was the highest (*p *< 0.022) in LE and GP. The ADF digestibility in the citrus by-products varied between 72.30 for OP and 68.10 g per 100 g of DM for LE (*p *< 0.034).

**Table 3:** Total gas production volume, specific rates of digestion and lag phase of the whole, neutral detergent soluble carbohydrate, neutral detergent fiber, acid detergent fiber, hemicellulose in four citrus by-products.

	**Total gas production volume ** **(mL per 100 mg DM)**	**Specific rates of digestion ** **(per hr)**	**Lag phase ** **(hr)**
**Grapefruit pulp**			
**Whole (based on DM)**	44.39^a^	0.068^b^	1.03^c^
**Neutral detergent soluble carbohydrate** 1	39.63^b^	0.077^a^	1.13^c^
**Neutral detergent fiber**	5.36^c^	0.026^d^	1.93^b^
**Acid detergent fiber**	1.92^e^	0.046^c^	0.72^d^
**Hemicellulose ** 2	3.73^d^	0.018^e^	3.63^a^
**SEM**	5.04	0.0061	0.32
***p*** **-value**	0.001	0.001	0.001
**Lemon pulp**			
**Whole (based on DM)**	43.60^a^	0.065^a^	1.06^b^
**Neutral detergent soluble carbohydrate** 1	37.35^b^	0.081^a^	1.33^b^
**Neutral detergent fiber**	14.26^c^	0.007^b^	0.40^c^
**Acid detergent fiber**	8.67^d^	0.007^b^	0.31^c^
**Hemicellulose ** 2	5.69^d^	0.007^b^	3.33^a^
**SEM**	4.174	0.0090	0.30
***p*** **-value**	0.001	0.001	0.001
**Lime pulp**			
**Whole (based on DM)**	44.04^a^	0.095^b^	0.43^b^
**Neutral detergent soluble carbohydrate** 1	40.21^b^	0.108^a^	0.56^b^
**Neutral detergent fiber**	5.38^c^	0.018^d^	0.53^b^
**Acid detergent fiber**	4.16^c^	0.013^e^	0.30^c^
**Hemicellulose ** 2	1.65^d^	0.028^c^	3.43^a^
**SEM**	5.05	0.010	0.33
***p*** **-value**	0.001	0.001	0.001
**Orange pulp**			
**Whole (based on DM)**	40.96^a^	0.053^b^	0.70^b^
**Neutral detergent soluble carbohydrate** 1	35.76^b^	0.062^a^	0.93^b^
**Neutral detergent fiber**	8.12^c^	0.011^cd^	0.96^b^
**Acid detergent fiber**	8.01^c^	0.005^d^	0.21^c^
**Hemicellulose ** 2	2.59^d^	0.020^c^	2.60^a^
**SEM**	4.27	0.006	0.26
***p*** **-value**	0.001	0.001	0.001

1 determined by subtraction method (whole minus neutral detergent fiber).

2 determined by subtraction method (Neutral detergent fiber minus acid detergent fiber.

**abcde:** Different superscripts indicate significant differences within each column (*p* < 0.05).

## Discussion

The DM and CP concentrations in this study were lower than those reported by Martínez-Pascual and Fernândez-Carmona for LP samples.^19^ Grapefruit pulp had higher ADF and lower ash, however, CP content was similar to that reported by Scerra *et al*.^20^ The variation in nutrient composition of citrus by-products could be attributed to several factors, including source of fruit and type of processing. Bampidis and Robinson compared chemical composition of different source of citrus by- products and who found that there were remarkable differences among chemical compositions of different source of citrus fruit and processing method.^21^ Drying of citrus pulp above 130 ˚C, the ADF, lignin and DM losses increase 2.00 to 2.50% for each additional 10 ˚C. It is evident that heating proteins can reduce their nutritive value due to Maillard polymerization.^19^

The single-pool equation used to model the data contains three parameters representing pool size (expressed as gas volumes), specific rate (maximum rate/maximum volume), and a single lag term.^22^ The results showed that NDSC fraction has major role in total gas production of citrus by-products. The NDSC and ADF from citrus by-products showed a short lag time in gas production. Lag time for NDF was higher than ADF. Also, there was long lag time for hemicelluloses, as a result higher lag time observed for NDF compared to ADF may due to lag time of hemicelluloses fraction. 

For all citrus by-products specific rate of NDSC were lower than the whole test feed with the exception of LE (Table 3). The presence of NDSC in the whole test feed might decrease the specific rate of carbohydrate fraction of whole test feed as compared with the NDSC fraction. In agreement with these finding, Miron *et al*. showed that inclusion of soluble carbohydrate reduced the rate of alfalfa hay cell wall degradation by a mixed rumen population.^23^ Similarly, Arroquy *et al*. reported that the adverse effect of supplemental non fiber carbohydrates were most readily evident in the form of a depression in the fractional rate of disappearance.^24^ In contrast, the specific rate of whole LE was unaffected by NDSC fraction. 

For GP, LI and OP specific rate of NDF were lower than the ADF. It can infer that presence of hemicelluloses in the NDF fraction resulted in a significant depression in the rate of NDF fraction as compared to the ADF fraction. This effect might be due to the hemicelluloses–fermenting bacteria competing with the NDF-digesting bacteria for available N, and that the inclusion of adequate quantities of rumen degradable protein in the diet might prevent hemicelluloses from decreasing NDF digestibility.^25^

For test feeds, lag time of ADF was lower than the NDF with the exception of LE. These results showed the possible involvement of hemicelluloses in the attachment mechanism of the cellulolytic bacteria and it can infer that the hemicelluloses–fermenting bacteria cause to delay in adhesion of cellulolytic bacteria. The same effect of carbohydrate fractions on bacterial adhesion was reported by Minato *et al*.^26^ These investigators reported that the adhesion of *Fibrobacter succinogenes* to cellulose is strongly inhibited by 1.00% cellobiose. Such conditions might be involved in the changes in the lag time of NDF which were observed in our experiments. It can be considered that this effect of hemicelluloses on the colonization of fiber by cellulolytic bacteria may be important in NDF disappearance.

Schofield and Pell evaluated validation of subtraction method and reported that variance for subtraction curve such as the NDSC is the summation of the variances for the whole feed and NDF measurements and there is a relatively high cumulative error in this curve.^7^ However, this error does not exhibit a problem for feed such as citrus by-products where the NDSC content is relatively high. Result of kinetics digestion showed that 1) the pool size of the rapidly digesting fraction was high for all citrus by-products; 2) there were no similarity between the specific rates of the whole citrus by-products; 3) the rate for the digesting of NDSC fraction was higher than the same fraction of the whole test feed; 4) lag times for feed fraction were varied. These differences in lag time represented the largest quantitative shift between the whole tested feed and NDF.

There was no significant difference for gas production (A) between citrus by-products. The amount of gas production for OP was in agreement with Cone *et al*. who reported that gas production was 36 mL per 100 mg OM.^27^ Overall, in the current study high amounts of total gas production volume (A) in four citrus by-products might be due to high contents of fermentable carbohydrate and energy. It has been suggested that gas production shows high content of digestible energy.^28^ Specific rate of whole (c) was the highest in LP compared to the other by-products. The ranking of fractional rate of whole citrus by-products in descending order was LP, GP, LE and OP. Our results showed that whole citrus by-products contain higher specific rate of digestion than the corn grain (0.048 per hr).^29^ The high gas production and specific rates of digestion can be due to the high amounts of soluble carbohydrate and soluble fiber. Lashkari and Taghizadeh evaluated carbohydrate fractionation of citrus by-products and reported that more than 35.00% of carbohydrate was consisted of soluble carbohydrate that can be fermented easily and rapidly.^30^ The same authors reported that citrus by-products contain more than 36.00% of soluble fiber such as pectin and also, this soluble fiber can be utilized by rumen microorganism as a source of fermentable energy. Unfortunately, there are no previously published data for specific rates of digestion and total gas production volume of citrus by-products including GP, LE and LP.

Aregheore using sheep fed by dried OP reported that the apparent digestibility of DM in OP was 84.10 g per 100 g DM.^31^ Dry matter digestibility of OP in current experiment was higher than that published previously.^31^ This could be expected, because Aregheore determined DM digestibility by *in vivo* method and reported that OP have high DM digestibility.^31^ The result of the current study demonstrated that DM of citrus by-products possessed high rumen degradability. Neutral detergent fiber digestibility was the highest in LE and GP. In agreement with these findings Hall *et al*. reported NDF digestibility in dried OP was 75 g per 100 g DM.^6^ The NDF fraction of citrus by-products was more extensively digested in our study (Table 2). This result was in agreement with Miron *et al*. who found that NDF of citrus by-products contains high potential for degradability in the rumen.^32^ For LP (69.10 g per 100 g DM) and LE (68.10 g per 100 g DM), ADF digestibility was lower than the other tested feeds. Results of the current study were inconsistent with those reported by Brown and Johnson who found that ADF digestibility of OP was 82.10 g per 100 g DM.^33^ Discrepancies among studies may be explained by the differences in processing effects of citrus by-products. The high gas production and DM digestibility observed in the current experiment may be due to their high NDF and ADF digestibility (Table 2). The high DM digestibility of citrus by-products as determined by *in vitro* methods may be the result of their low cell wall content (Table 1). Generally, all citrus by-products have high degradability potential and consequently DM content of citrus by-products can be widely degraded in the rumen (Table 2). Unfortunately, there are no previously published data for DM, NDF and ADF digestibility of citrus by-products including GP, LE and LP.

In conclusion, the curve-subtraction method allowed the assessment of the digestion kinetics of the NDSC and hemicelluloses fractions in feedstuffs. These methods offer analytical tools to define carbohydrate fractions to be used in nutritional models of ruminant diets. Citrus by-products present a potential appropriate source of degradable carbohydrate fractions which may be appropriate source of the energy for ruminant nutrition. It can be concluded that the presence of readily available NDSC affect fractional rate and lag time of whole test feed. Therefore, it is important to consider the interactions among the carbohydrate fraction, as it aids in formulating diets for maximal ruminal DM and NDF disappearance.
